# How does graphotactic knowledge influence children's learning of new spellings?

**DOI:** 10.3389/fpsyg.2013.00701

**Published:** 2013-10-04

**Authors:** Sébastien Pacton, Amélie Sobaco, Michel Fayol, Rebecca Treiman

**Affiliations:** ^1^Laboratoire Mémoire et Cognition, Psychology Department, Université Paris DescartesBoulogne Billancourt, France; ^2^Institut Universitaire de FranceParis, France; ^3^Psychology Department, LAPSCO, Université Blaise PascalClermont Ferrand Cedex1, France; ^4^Psychology Department, Washington University in St. LouisSt. Louis, MO, USA

**Keywords:** spelling, graphotacics, implicit learning, statistical learning, orthographic learning

## Abstract

Two experiments investigated whether and how the learning of spellings by French third graders is influenced by two graphotactic patterns: consonants cannot double in word-initial position (Experiment 1) and consonants cannot double after single consonants (Experiment 2). Children silently read meaningful texts that contained three types of novel spellings: no doublet (e.g., *mupile, guprane*), doublet in a legal position (e.g., *muppile, gupprane*), and doublet in an illegal position (e.g., *mmupile, guprrane*). Orthographic learning was assessed with a task of spelling to dictation. In both experiments, children recalled items without doublets better than items with doublets. In Experiment 1, children recalled spellings with a doublet in illegal word-initial position better than spellings with a doublet in legal word-medial position, and almost all misspellings involved the omission of the doublet. The fact that the graphotactic violation in an item like *mmupile* was in the salient initial position may explain why children often remembered both the presence and the position of the doublet. In Experiment 2, children recalled non-words with a doublet before a single consonant (legal, e.g., *gupprane*) better than those with a doublet after a single consonant (illegal, e.g., *guprrane*). Omission of the doublet was the most frequent error for both types of items. Children also made some transposition errors on items with a doublet after a single consonant, recalling for example *gupprane* instead of *guprrane*. These results suggest that, when a doublet is in the hard-to-remember medial position, children sometimes remember that an item contains a doublet but not which letter is doubled. Their knowledge that double consonants can occur before but not after single consonants leads to transposition errors on items like *guprrane*. These results shed new light on the conditions under which children use general knowledge about the graphotactic patterns of their writing system to reconstruct spellings.

## Introduction

All alphabetic writing systems are based on the principle that graphemes represent phonemes, but they vary in orthographic depth, from consistent mappings between phonemes and graphemes in shallow writing systems to inconsistent mappings in deep writing systems (Sprenger-Charolles, 2003; Ziegler and Goswami, 2005). The fact that there is often more than one possible spelling for a given phoneme in deep writing systems like English and French is a major source of difficulty in learning to spell (Seymour et al., 2003). In some cases, spellers must choose between representing a sound with a single letter or a doublet, that is, a sequence of two identical letters. This case is especially frequent in French, where there is usually no phonological distinction between single and double consonants. For example, *ule* and *ulle* are similarly pronounced in *bulle* (“bubble”) and *formule* (“formula”), and *acro* and *accro* are similarly pronounced in *acrobate* (“acrobat”) and *accrocher* (“to hang up”). French children and adults sometimes omit a required doublet (e.g., *bulle* misspelled as *bule*) or double a letter when not required (e.g., *formule* misspelled as *formulle*; Lucci and Millet, 1994; Manesse et al., 2007).

It is often assumed that, when there is more than one possible spelling for a phoneme, spellers must memorize which one is correct. However, that may not always be true. French, like other writing systems, has statistical patterns that reduce or sometimes even eliminate the need for rote word-by-word memorization (e.g., Treiman et al., 2002; Deacon et al., 2008). Concerning consonant doubling, French spellers can rely on various graphotactic regularities, that is, statistical patterns concerning the arrangement of letters in words. These include the fact that some consonants double more often than others (e.g., *l* frequently doubles, *d* rarely doubles, and *k* never doubles) and that doublets may occur between two vowels (e.g., *nappe* “tablecloth”; *apporter “to bring”*) or between a vowel and a single consonant (e.g., *apprendre* “to learn”) but not at the beginnings of words or after a single consonant. Use of such graphotactic regularities cannot help spellers to avoid misspellings such as *nape* and *aprendre*, with omission of the doublet, or misspellings such as *formulle*, with *l* mistakenly doubled, but it can help them to avoid misspellings such as *nnape*, with an initial doublet, and *aprrendre*, with a doublet after a single consonant. However, reliance on graphotactic regularities in order to spell words that contain uncommon patterns should lead to the production of misspellings. These misspellings should not be random but should rather show a bias toward the most likely spellings. For example, French people who encounter a new word such as *gumadde* may subsequently spell it *gummade* if they remember that this word includes a doublet but not which letter is doubled and if they use their knowledge that *m* is more likely to double than *d*. In the present study, we tested these ideas in a study of French third graders. We asked whether and how children's learning of spellings is influenced by two graphotactic patterns that are not typically taught explicitly in French schools: consonants cannot double in word-initial position (Experiment 1) and after single consonants (Experiment 2).

Much of the evidence for children's knowledge of graphotactic patterns comes from studies in which they are asked to decide which of two non-words looks more like a word of their language. As early as first grade, for example, French children were more likely to choose non-words including doublets for pairs like *imose* and *immose*, where *m* is frequent in both single and double formats, than for pairs like *idose* and *iddose*, where *d* is frequent as a single letter but rarely doubles in French (Danjon and Pacton, 2009; see also Cassar and Treiman, 1997; Pacton et al., 2001). In judgment tasks such as these, graphotactic knowledge is the only source of information on which participants can rely. Here we went beyond previous studies by examining the influence of graphotactics in a more natural situation in which children can also use memory for word-specific spellings. We used, in addition to a judgment task, a task that models the everyday situation in which children encounter a new word during the course of reading for meaning and later try to spell it. Such a situation has been used in a number of other studies whose results suggest that, to a large extent, people teach themselves to spell through reading (Burt and Butterworth, 1996; Share, 1999, 2004; Burt and Furry, 2000; Nation et al., 2007).

Wright and Ehri (2007) reported an influence of graphotactics on memory for spellings in U.S. kindergartners and first graders. In their study, children were taught to pronounce isolated words. Children saw items that included only single consonants (e.g., *rug*), items that began with consonant doublets (e.g., *rrug*), and items that ended with consonant doublets (e.g., *rugg*). In English, consonant doubling virtually never occurs at the beginnings of words (*llama* is the chief exception, but this is not a word that young children would know). However, doubling sometimes occurs at the ends of words (e.g., *ball, miss*). The children studied by Wright and Ehri learned to read words with final doubled consonants as easily as words with only single consonants, but it took them longer to learn to read words with initial doublets. When children were later asked to spell the words, they remembered final doublets almost as well as final singletons. However, their memory for initial doublets was very poor. Many children misremembered words containing initial doublets without any doublets (e.g., *rrug* as *rug*), but a number of children doubled the final consonant instead of the initial one (e.g., *rrug* as *rugg*). Children rarely erroneously doubled final consonants when an item like *tub* had been taught, suggesting that a memory that some letter was doubled in *rrug* caused them to produce the error *rugg*. Children's knowledge of graphotactic patterns apparently led them to double the consonant in a legal rather than an illegal position.

Wright and Ehri's (2007) finding that kindergartners and first graders remembered final doubled letters better than initial doubled letters contrasts with the often reported finding that children pay special attention to initial letters in words (Ehri and Saltmarsh, 1995; Bowman and Treiman, 2002; Treiman et al., 2007) and remember them better than subsequent letters (e.g., Mendenhall, 1930; Jensen, 1962; Kooi et al., 1965; Stage and Wagner, 1992; Treiman et al., 1993). Wright and Ehri's finding also contrasts with the common finding that people recall distinctive items more easily than common items (e.g., Hunt and Worthen, 2006; Gounden and Nicolas, 2012). This facilitative effect has been shown in memory tasks using bizarre images and sentences describing bizarre events, as well as in memory tasks for words that contain atypical letter combinations. For example, Zechmeister (1972) showed that adults were better at remembering that they had seen words containing atypical letter combinations than words containing more typical letter combinations. Similarly, Grainger and Ziegler (2011) suggested that letter combinations that are encountered infrequently in other words are highly informative with respect to word identity.

In Experiment 1, we asked whether and how the learning of spellings by French third graders is influenced by their knowledge that consonants cannot double in word-initial position. Experiment 1 differed from the study of Wright and Ehri (2007) not only in the language under study and the age of the children but also in several other dimensions. One difference is that Wright and Ehri used non-words with doublets either at the beginning or at the end. Because French consonants may double in internal position but not in initial and final positions, we used spellings without doublets (e.g., *mupile*), spellings with an initial doublet (e.g., *mmupile*), and spellings with a medial doublet (e.g., *muppile*). The three types of non-word spellings all had the same pronunciations. Another difference is that, while Wright and Ehri (2007) presented children with spellings of isolated words and explicitly taught the children how to pronounce them, we included a task in which children encountered a new word during the course of reading for meaning and were later asked to spell it. In Experiment 1, children were exposed to the three types of non-word spellings just mentioned, with frequency of exposure equated, in the context of meaningful texts. Children were not explicitly asked to learn the new spellings. Memory for the spelling of non-words was tested after children had finished reading the stories in which the non-words were embedded and performing a filler task. We also included a judgment task like those of previous studies in which children saw pairs of non-words (e.g., *nummar* and *nnumar*) and judged which one looked more like a word of their language (Cassar and Treiman, 1997; Wright and Ehri, 2007; Danjon and Pacton, 2009).

If children show the same kind of pattern that Wright and Ehri (2007) reported, they should produce fewer correct spellings of non-words containing an initial doublet (e.g., *mmupile*) than of those containing a medial doublet (e.g., *muppile*) or those not containing a doublet (e.g., *mupile*). Both omission and transposition errors should be committed on items with an initial doublet, but omission errors should be largely restricted to items with a medial doublet. Thus, transposition of the doublet from one position to another should be more common for items with an initial doublet (as when *mmupile* is remembered as *muppile*) than for items with a medial doublet (as when *muppile* is remembered as *mmupile*). In contrast, if children's attention is captured by the presence of a doublet at the beginning of an item, and if they remember the initial part of the item well, they may often correctly recall non-words with an initial doublet. Children might even be more accurate on non-words with initial doublets than on non-words with medial doublets. The first graders studied by Wright and Ehri (2007) did not show this pattern, perhaps because their knowledge that initial consonants cannot double was relatively weak. Third graders may be more knowledgeable that items such as *mmupile* are orthographically odd. That orthographic distinctiveness might capture their attention and help them to remember not only the presence of the doublet but also its position.

## Experiment 1

### Methods

#### Participants

The participants were 24 native speakers of French from an elementary school in Poitiers, France. The children were tested at the end of their third grade year. Their characteristics in terms of age, gender, and spelling ability are presented in Table 1.

**Table 1:** **Characteristics (age and gender) and scores on the general spelling ability and judgment tasks (standard deviations in parentheses)**.

**Characteristics and tests**	**Experiment**	
	**Experiment 1**	**Experiment 2**
	**(***N*** = **24**)**	**(***N*** = **24**)**
Age (in years)	8.83 (.32)	9.00 (0.40)
Gender (female, male)	12, 12	15, 9
General spelling ability (maximum = 50)^a^	36.75 (6.35)	36.00 (7.56)
Judgment task (% correct)		
Consonants cannot double in word-initial position (selection of *nummar* rather than *nnumar*)	90.63 (11.21)	94.79 (7.29)
Consonants cannot double after a single consonant (selection of *apprulir* rather than *aprrulir*)	72.40 (17.28)	75.52 (20.35)
Only some consonants can double (selection of *onnave* rather than *ojjave*)	86.46 (14.71)	89.06 (10.63)

a Average score for third graders on this test is 32.38. The scores on the general spelling ability test were not significantly different for participants in Experiments 1 and 2 [t_(46)_ = 0.37, p = 0.71].

#### Stimuli

***Non-word learning task.*** We constructed six bisyllabic non-words that were phonologically legal in French: /fonεs/, /mypil/, /nifyd/, /pamεv/, /ritod/, and /taryl/. Each non-word included two target consonants that are often spelled with a double letter in French: /f/, /m/, /n/, /p/, /r/, and /t/. Each target consonant was the onset of the first syllable in one non-word and the onset of the second syllable in another. We created no-doublet, initial-doublet, and medial-doublet spellings of each non-word, as in *mupile* (no doublet), *mmupile* (initial doublet), and *muppile* (medial doublet). The specific rendition of each non-word was counterbalanced across children so that (1) each child saw two no-doublet spellings, two initial-doublet spellings, and two medial-doublet spellings; (2) each child saw only one spelling of a given non-word (e.g., only the no-doublet spelling *mupile* for a child and only the medial-doublet *muppile* for another child); and (3) each spelling of a given non-word was presented to eight children (e.g., the no-doublet spelling *mupile* to eight children and the medial-doublet *muppile* to eight other children).

We created three stories, with an average length of 157 words (range 153–167), in which we embedded two non-words. The non-words served as nouns, for example the name of a type of fruit. Two non-words of the same type (i.e., no, initial, or medial doublet) were not included in the same story. A sample story appears in the Appendix. Each non-word occurred five times in each story. Four questions were prepared about each story. The first required participants to select an appropriate title for the story from a list of three. The next three questions were true/false questions about the content. The order of the stories and the non-words embedded in them were randomized across subjects.

***Judgment task.*** This task included 24 pairs of non-words, which are shown in the Appendix. In the eight pairs used to assess children's knowledge that consonants cannot double in word-initial position, one non-word included a medial doublet and the other an initial doublet, as in the pair *nummar* and *nnumar*. The two doublets were formed with consonants that often double in French. In eight pairs used to assess children's knowledge that consonants cannot double before a consonant, one non-word included a doublet before a single consonant and the other a doublet after a single consonant, as in *apprulir* and *aprrulir*. The two doublets were formed with consonants that can double in French. In eight pairs used to assess children's knowledge that only some consonants can double, one non-word included a frequent doublet in word-medial position and the other a doublet formed with a consonant that never doubles in French, also in word-medial position, as in the pair *onnave and ojjav*. For each type of pair, legal non-words were on the right in half of the trials and on the left in the other half. They were arranged in a random order and stapled together in order to make a booklet. The booklet started with three practice pairs in which only one item could be a French word.

***General spelling ability.***General spelling ability was assessed with the Corbeau standardized spelling subtest (Chevrier-Muller et al., 1997). This test provides a global spelling score which reflects children's ability to produce spellings that are phonologically plausible, even though not necessarily orthographically correct, children's use of word-specific spelling knowledge, and children's correct use of grammatical markers. We used this test to ensure that the spelling ability of the participants was representative of the expected level of French third graders and that it was similar for the participants in Experiments 1 and 2.

#### Procedure

Children were tested as a group. The first day, children were told that they would receive booklets that included stories along with questions about each. Children were asked to read one story silently and move to the next page to answer questions about it, without rereading the story, then go to the next story, and so on. After this, children performed a letter cancellation task for 10 min. Then, the experimenter pronounced each of the six non-words and asked children to spell them as written in the texts they read. The experimenter explicitly informed children that they should not copy each other's spellings because they did not read the same texts. Furthermore, the presence of two adults in the classroom, the experimenter and children's teacher, allowed us to keep an eye on children.

The next day, the standardized spelling subtest was given by the experimenter. The judgment task was then given. Children were told that the experimenter had made up new words that no one had ever seen or heard before and that they would have to decide which made-up word is more like words they know. They were asked to look at the first practice pair and to circle that item on their booklet. This procedure was repeated with the remaining two practice items. Children received feedback for the practice items. They then went on to the test items, and here they were not told whether their responses were correct or incorrect.

### Results

#### Non-word learning task

For each participant, we counted the number of spellings that contained only single consonants, the number of spellings that contained an initial doublet, and the number of spellings that contained a medial doublet. The mean values for the 24 participants, transformed into percentages, are shown in Table 2. Correct spellings, defined as those in which both target consonants (e.g., *m* and *pp* for the item *muppile*) were spelled as in the story, were more common on no-doublet items than on items presented with a doublet. Among the doublet items, correct spellings were more common for initial- than for medial-doublet items, as Table 2 shows. Analyses of variances (ANOVAs) on the number of correct spellings using subjects (*F*_1_) and items (*F*_2_) as random variables confirmed the main effect of item type [*F*_1(2, 46)_ = 11.66, *p* < 0.001, η^2^_*p*_ = 0.34; *F*_2(2, 10)_ = 41.50, *p* < 0.001, η^2^_*p*_ = 0.89]. Planned comparisons revealed significantly more correct spellings for no-doublet items (83.33%) than for the two types of doublet items [*F*_1(1, 23)_ = 17.73, *p* < 0.001, η^2^_*p*_ = 0.44; *F*_2(1, 5)_ = 103.91, *p* < 0.001, η^2^_*p*_ = 0.95] and significantly more correct spellings for initial- than for medial-doublet items [60.42 vs. 39.58%, *F*_1(1, 23)_ = 5.37, *p* = 0.03, η^2^_*p*_ = 0.19; *F*_2(1, 5)_ = 14.81, *p* = 0.01, η^2^_*p*_ = 0.75].

**Table 2:** **Percentage of different types of spellings produced in Experiment 1**.

**Type of spelling produced**	**Type of spelling presented**		
	**No doublet**	**Medial doublet**	**Initial doublet**
No doublet	**83.33 (28.23)**	54.17 (38.78)	29.17 (29.18)
Medial doublet	6.25 (22.42)	**39.58 (32.90)**	*6.25 (16.89)*
Initial doublet	6.25 (16.89)	*6.25 (16.89)*	**60.42 (25.45)**
Other	4.17 (14.12)	0.00 (0.00)	4.17 (14.12)

Correct spellings are in bold and transposition errors in italics; standard deviations are in parentheses.

Other spellings included phonologically incorrect renditions of one of the target consonants.

We defined omission errors as those in which a doublet consonant was spelled with a singleton (e.g., *mmupile* or *muppile* misspelled as *mupile*). Transposition errors were defined as those that involved movement of the doubling feature to the wrong target consonant: the medial consonant instead of the initial consonant for the initial-doublet items (e.g., *mmupile* misspelled as *muppile*) and the initial consonant instead of the medial consonant for the medial-doublet items (e.g., *muppile* misspelled as *mmupile*). Misspellings of doublet items almost always involved omission of the doublet. There were only six transposition errors produced by three children, three on initial-doublet items and three on medial-doublet items.

#### Judgment task

Table 1 shows the scores in the test of graphotactic knowledge. Children showed very good knowledge that consonants cannot double in word initial position and that only some consonants can double. There was also evidence that children knew that double consonants can occur before but not after single consonants, although their knowledge about this property appeared to be less well-established. *T*-tests using subjects (*t*_1_) and items (*t*_2_) as random variables showed that the selection rate of legal items was significantly above chance (50%) for each of the three properties [*t*_1_s_(23)_ > 6.35, *p*s < 0.001; *t*_2_s_(7)_ > 9.51, *p*s < 0.001]. An ANOVA showed that the number of correct responses varied as a function of the type of graphotactic property [*F*_1(2, 46)_ = 15.86, *p* < 0.001, η^2^_*p*_ = 0.41; *F*_2(2, 21)_ = 10.92, *p* < 0.001, η^2^_*p*_ = 0.51]. Planned comparisons revealed lower scores for items assessing knowledge that double consonants can occur before but not after single consonants than for items assessing the two other properties [*F*_1(1, 23)_ = 25.61, *p* < 0.001, η^2^_*p*_ = 0.53; *F*_2(1, 21)_ = 20.79, *p* < 0.001, η^2^_*p*_ = 0.50], with no difference between items assessing which consonants can double and items assessing the fact that consonants cannot double in word-initial position (*p*s > 0.19).

### Discussion

French third graders were better at recalling spellings that did not include doublets (e.g., *mupile*) than spellings that did. Among items with doublets, children performed better on those with an initial doublet (e.g., *mmupile*) than those with a medial doublet (e.g., *muppile*). These differences were found even though the children had seen the three types of spellings equally often in the texts they read. Transposition errors were rare (6 of 96 spellings produced), whether the doublet was positioned legally or illegally. Almost all of the misspellings involved omitting one consonant of a doublet.

The pattern of results in Experiment 1 is quite different from that found by Wright and Ehri (2007) with U.S. first graders. The children in that study produced fewer correct spellings for items with an initial doublet than for items with a final doublet, and they made transposition errors on initial-doublet items. However, our finding that items with a medial doublet were more often misspelled than items with an initial doublet fits well with other findings showing that misspellings are more common in the middles of words than at the beginnings or ends (e.g., Jensen, 1962; Stage and Wagner, 1992; Treiman et al., 1993).

The first graders tested by Wright and Ehri (2007) scored above chance, but not far above (67% correct), in choosing between a spelling with a doublet in the initial (illegal) position and a spelling with a doublet in the final (legal) position. The third graders of the present study chose spellings including a doublet in medial (legal) position far more often than spellings including a doublet in initial (illegal) position (91%). This confirms previous findings (e.g., Danjon and Pacton, 2009) that, in grade 3, French children's knowledge that doublets are illegal in word-initial position is well-established. Given this firmly established graphotactic knowledge, items like *mmupile* that include a graphotactic violation in initial position are orthographically distinctive for third graders. This orthographic distinctiveness may have captured third graders' attention. This helped them to remember both the presence and the position of the doublet, potentially explaining why they rarely transposed the doublet from the initial to the medial position.

Of course, there are several other reasons why the results of Experiment 1 may have differed from those reported by Wright and Ehri (2007). For example, the spellings were presented in isolation in Wright and Ehri's study but were embedded within stories in our study, the learning situation was intentional in Wright and Ehri's study but incidental in our study, and spellings were presented in uppercase letters in Wright and Ehri's study but in lowercase letters in our study. We return to this issue in the General Discussion.

Regardless of the potential role of these other variables, if the position of the graphotactic illegality, third graders' strong knowledge of this illegality, or both, capture children's attention, then children of the same age and spelling level should exhibit a pattern of performance more similar to that reported by Wright and Ehri (2007) for a graphotactic violation located in word-medial position, which is less likely to capture their attention. We investigated this issue in Experiment 2 by exploring whether French third graders' learning of spellings was influenced by their knowledge that consonants can double before, but not after, single consonants. Experiment 2 used the same procedure as Experiment 1, except that we modified the type of items embedded in the stories. Specifically, we used spellings without doublets (e.g., *guprane*), spellings with a doublet before a single consonant (e.g., *gupprane*), and spellings with a doublet after a single consonant (e.g., *guprrane*). The results of Experiment 1 and of Danjon and Pacton (2009) show that French third graders are sensitive to illegality of items like *gupprane*, but not as much as to the illegality of doublets in word-initial position. In Experiment 2, where the graphotactically illegal item is less distinctive and where all targets are located in the middles of the items, we expected that children would recall the legal spellings *guprane* and *gupprane* better than the illegal spelling *guprrane* and that they would sometimes spell the last as *gupprane*, transposing the doubling from an illegal to a legal position.

## Experiment 2

### Methods

#### Participants

The participants were 24 native speakers of French from an elementary school in Poitiers, France. The children were tested at the end of their third grade year. Table 1 provides information about their age, gender, and spelling ability.

#### Stimuli

***Non-word learning task.*** We constructed six phonologically legal bisyllabic non-words: /dyflin/, /gypran/, /mifr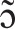/, /nokril/, /toplir/, and /viklar/. Each non-word included a consonant cluster at the beginning of the second syllable. We created AB, AAB, and ABB spellings of each non-word, as in *guprane* (AB), *gupprane* (AAB), *and guprrane* (ABB). As in Experiment 1, the specific rendition of each non-word was counterbalanced across children so that (1) each child saw two AB spellings, two AAB spellings, and two ABB spellings; (2) each child saw only one spelling of a given non-word; and (3) each spelling of a given non-word was presented to eight children.

The non-words were embedded in the same stories as in Experiment 1, with the same constraints that two non-words were included in each story and that two non-words of the same type (i.e., AB, AAB, or ABB) were not included in the same story. Also as in Experiment 1, each non-word occurred five times in each story and the order of the stories and the non-words embedded in them were randomized across subjects. We used the same judgment task and general spelling test as in Experiments 1 and 2.

#### Procedure

The procedure was identical to that of Experiment 1.

### Results

#### Non-word learning task

For each participant, we counted the number of spellings that contained only single consonants, the number of spellings that contained doublet before a single consonant, and the number of spellings that contained a doublet after a single consonant. Table 3 shows the mean values for the 24 participants, transformed into percentages. Correct spellings were more common for AB items than for items with a doublet, and among these items, correct spellings were more common for AAB (legal) than for ABB (illegal) items. ANOVAs on the number of correct spellings using subjects and items as random variables confirmed the main effect of item type [*F*_1(2, 46)_ = 27.78, *p* < 0.001, η^2^_*p*_ = 0.55; *F*_2(2, 10)_ = 37.70, *p* < 0.001, η^2^_*p*_ = 0.88]. Planned comparisons revealed significantly more correct spellings for AB items (87.5%) than for AAB and ABB items [*F*_1(1, 23)_ = 39.66, *p* < 0.001, η^2^_*p*_ = 0.63; *F*_2(1, 5)_ = 148.00, *p* < 0.001, η^2^_*p*_ = 0.97] and significantly more correct spellings for AAB than ABB items [52.1 vs. 14.6%, *F*_1(1, 23)_ = 15.15, *p* < 0.001, η^2^_*p*_ = 0.40; *F*_2(1, 5)_ = 13.08, *p* = 0.015, η^2^_*p*_ = 0.72].

**Table 3:** **Percentages of AB, AAB, and ABB spellings produced in Experiment 2 as a function of type of spelling presented**.

**Type of spelling produced**	**Type of spelling presented**		
	***AB***	***AAB***	***ABB***
*AB*	**87.50 (26.58)**	41.67 (40.82)	50.00 (32.97)
*AAB*	8.33 (24.08)	**52.08 (42.93)**	*25.00 (32.97)*
*ABB*	2.08 (10.21)	*2.08 (10.21)*	**14.58 (23.22)**
Other doublets	2.08 (10.21)	4.17 (14.12)	6.25 (22.42)
Other spellings	0.00 (0.00)	0.0 (0.0)	4.17 (14.12)

Correct spellings are in bold and transposition errors in italics; standard deviations are in parentheses.

AB spelling: the two consonants in the target cluster were single; AAB spellings: only the first consonant of the target cluster was doubled; ABB spellings: only the second consonant of the target cluster was doubled; Other doublets: the consonant that was doubled did not belong to the target cluster (e.g., guprane, gupprane or guprrane misspelled as gupranne). Other spellings: one of the consonants in the target cluster was spelled in a phonologically illegal manner.

Omission errors were those in which a doublet consonant in AAB or ABB item was spelled with a singleton (e.g., *gupprane* or *guprrane* misspelled as *guprane*). Transposition errors involved movement of the doubling feature to the wrong target consonant: the first instead of the second consonant of the cluster for ABB items (e.g., *guprrane* misspelled as *gupprane*) and the second instead of the first consonant of the cluster for AAB items (e.g., *gupprane* misspelled as *guprrane*). Whereas omission errors were as frequent for AAB as for ABB items (41.7% and 50.0%, respectively), transposition errors were restricted to ABB items. Indeed, ten children made one (*n* = 8) or two (*n* = 2) transposition errors on ABB items, a rate of 25.0% transposition errors, whereas only one child made one transposition error on an AAB item (2.1%). While omission errors were equally common for AAB and ABB items [*t*_1(23)_ = 0.81, *p* = 0.42; *t*_2(5)_ = 0.21, *p* = 0.84], transposition errors were more common for ABB than AAB items [*t*_1(23)_ = 3.11, *p* = 0.005; *t*_2(5)_ = 4.00, *p* = 0.010].

Importantly, the larger number of transposition errors on ABB items than AAB items did not reflect a general trend to double the first consonant of the consonant cluster, irrespective of the type of item that was presented in the text. Indeed, children produced AAB spellings for ABB items both more often than for AB items and less often than for AAB items. An ANOVA on the number of AAB spellings revealed a main effect of item type (AB, AAB, and ABB) [*F*_1(2, 46)_ = 14.71, *p* < 0.001, η^2^_*p*_ = 0.39; *F*_2(2, 10)_ = 14.11, *p* = 0.001, η^2^_*p*_ = 0.74]. Planned comparisons showed that AAB spellings were more common for AAB items than for AB and ABB items [*F*_1(1, 23)_ = 10.15, *p* = 0.004, η^2^_*p*_ = 0.31; *F*_2(1, 5)_ = 8.11, *p* = 0.036, η^2^_*p*_ = 0.62] and that AAB spellings were more common for ABB items than for AB items [*F*_1(1, 23)_ = 5.41, *p* = 0.029, η^2^_*p*_ = 0.19; *F*_2(1, 5)_ = 6.86, *p* = 0.047, η^2^_*p*_ = 0.58].

#### Judgment task

Table 1 shows the scores in the judgment task used to assess children's graphotactic knowledge. The scores were very similar to those reported in Experiment 1. In particular, although children had knowledge about all three of the graphotactic properties assessed, their knowledge that consonants cannot double in word-initial position and that only some consonants can double appeared to be better established than their knowledge that consonants cannot double after single consonants. The selection rate of legal items was significantly above chance (50%) for each of the three properties [*t*_1_s_(23)_ > 6.14, *p*s < 0.001; *t*_2_*s*_(7)_ > 7.76, *p*s < 0.001]. However, the number of correct responses varied as a function of the type of graphotactic property [*F*_1(2, 46)_ = 17.63, *p* < 0.001, η^2^_*p*_ = 0.43; *F*_2(2, 21)_ = 7.39, *p* = 0.004, η^2^_*p*_ = 0.41]. Planned comparisons revealed lower scores for the items assessing knowledge that double consonants can occur before but not after single consonants than for the items assessing the two other properties [*F*_1(1, 23)_ = 20.27, *p* < 0.001, η^2^_*p*_ = 0.47; *F*_2(1, 21)_ = 13.54, *p* = 0.001, η^2^_*p*_ = 0.39]. The scores tended to be higher for items assessing the fact that consonants cannot double in word-initial position than for items assessing which consonants can be doubled, although this result was significant only by participants [*F*_1(1, 23)_ = 7.27, *p* = 0.012, η^2^_*p*_ = 0.24; *F*_2(1, 21)_ = 1.24, *p* = 0.28, η^2^_*p*_ = 0.06]. Finally, analyses comparing the scores of the participants in the two experiments for each of the three properties revealed no significant experiment effect (all *p*s > 0.13).

### Discussion

French third graders were better at recalling spelling that did not contain doublets (e.g., *guprane*) than spellings that did. Among items with doublets, children showed better recall of those with a doublet before a single consonant (e.g., *gupprane*), which is legal in French, than those with a doublet after a single consonant, which is illegal in French (e.g., *guprrane*). Omission errors were the most frequent error for the two types of spellings containing doublets, and their frequency did not differ significantly according to whether the doublets were located in a legal (AAB items) or illegal position (ABB items). There were also some transposition errors, which were almost restricted to ABB items. The relatively frequent occurrence of transposition errors on ABB items, their uncommonness on AAB items, and the rare use of AAB spellings for AB items suggest that children sometimes remembered the presence of doubling but not the specific letter that was doubled. When this happened, children probably reconstructed a spelling based on their knowledge of which letters are most likely to double and in which positions. This reconstruction yielded correct spellings for AAB items but transposition errors for ABB items.

## General discussion

In two experiments, we investigated whether and how the learning of spellings by French third graders is influenced by two graphotactic patterns: consonants cannot double in word-initial position (Experiment 1) and consonants cannot double after single consonants (Experiment 2). To do this, we used an incidental orthographic learning task (Burt and Butterworth, 1996; Share, 1999, 2004; Burt and Furry, 2000; Nation et al., 2007). Children silently read meaningful texts that contained three types of novel spellings: those that did not contain any doublets (e.g., *mupile, guprane*), those with a doublet in a legal position (e.g., *muppile, gupprane*), and those with a doublet in an illegal position (e.g., *mmupile, guprrane*). Children were not instructed to remember the spellings, and their orthographic learning was subsequently assessed with a task of spelling to dictation.

In both experiments, children recalled items without doublets better than items with doublets. These results are consistent with previous findings about the difficulty of doublets for spellers of French (e.g., Manesse et al., 2007). This difficulty may arise, in part, because double consonants are less common than singletons. Children's difficulty in learning to spell items containing doublets may reflect the fact that spellings with common graphic patterns are easier to learn and remember than spellings with uncommon patterns.

Although the children in both experiments had more difficulty with doublets than singletons, other aspects of the results differed across the two experiments. In Experiment 1, children recalled spellings with an initial doublet (e.g., *mmupile*) better than spellings with a medial doublet (e.g., *muppile*). That is, they showed the surprising pattern of better performance on illegal than legal doublets. In Experiment 2, children recalled spellings with a doublet before a single consonant (e.g., *gupprane*) better than those with a doublet after a single consonant (e.g., *guprrane*). That is, they performed better on legal than illegal doublets. In both experiments, omission of the doublet was the most frequent error for both legal and illegal doublets. In Experiment 2, children also made some transposition errors on items with a doublet after a single consonant, recalling for example the legal *gupprane* instead of the illegal *guprrane*. Transpositions of a doublet from the illegal initial position to the legal medial position were uncommon in Experiment 1, however.

Why did the children in Experiment 1 perform better on illegal spellings like *mmupile* than on illegal spellings like *muppile*? The fact that illegality of an item like *mmupile* is in the salient initial position may be an important contributor. Children may have remembered the item with an illegal spelling pattern better than the item with a legal spelling pattern because the illegal pattern in the salient initial position captured their attention. It was only when we equated for position in Experiment 2, comparing legal and illegal spellings in the middles of the items, that we found better memory for legal spellings. This may help to explain why our results with initial doublets differed from those of Wright and Ehri (2007). The children in their study, who were kindergartners and first graders, had some knowledge about the illegality of items with initial doublets. However, this knowledge was not firmly established. The third graders in the present study had a strong knowledge that doublets cannot occur at the beginnings of words. This may have made the violation in items like *mmupile* very salient to them, helping them to remember both the presence and the position of the doublet.

Our third graders' relatively weak knowledge that consonants cannot double after single consonants may help to explain the transposition errors that we observed in Experiment 2. Although the children's graphotactic knowledge may have been sufficient in some cases to reconstruct the spelling of an item for which they remembered the presence of a doublet but not its position, it may have been insufficient to capture their attention when they read graphotactically illegal items like *guprrane*. Additional studies involving larger samples of participants who vary in literacy skill are needed to determine whether differences in the degree of graphotactic knowledge may explain why transposition errors are observed only for certain properties and/or only for certain individuals. Such studies should explore whether transposition errors are very rare among children who have a strong knowledge about a graphotactic property, as observed in Experiment 1, whereas biased transposition errors, in the direction of replacing illegal with legal spellings are made only by children who have an intermediate level of knowledge about a graphotactic property, as observed in Experiment 2. According to this view, children older than those involved in the present study or adults, who consistently express a preference for items such as *gupprane* over items such as *guprrane* in a judgment task would be expected to not commit transposition errors such as *gupprane* for *guprrane*. However, it is also possible that older children and adults would make such errors because, whereas the judgment task leads individuals to focus on the feature that distinguishes the non-words in a pair, namely the doublet either before or after the single consonant, reading may not require this level of analysis (e.g., Grainger and Whitney, 2004; Rayner et al., 2006).

We have suggested that the salience of the graphotactic violation in the initial position for third graders may help to explain why we found a different pattern of results in Experiment 1 than did Wright and Ehri (2007). However, there were a number of differences between our procedure and that of Wright and Ehri that could be important as well. One difference is that spellings were embedded in stories in our study but were presented in isolation in Wright and Ehri's study. We suspect that this is not a major reason why our Experiment 1 showed a different pattern of results than Wright and Ehri's experiment, however. Several studies have not found differences in orthographic learning according to whether spellings were seen in meaningful texts or in isolation (e.g., Cunningham, 2006; Nation et al., 2007; see also Wang et al., 2011, for the learning of items with regular spellings). Another difference is that the learning situation was incidental in our study but intentional in Wright and Ehri's study, and still another is that the items were presented in lowercase letters in our study but in uppercase letters in Wright and Ehri's study. Additional research is needed to determine whether these other factors are influential.

Although questions remain, our results show that learning to spell even in a deep orthography is not a matter of rote word-by-word memorization. By the end of third grade, French children show good knowledge of several important graphotactic patterns of their writing system. They appear to use these patterns to learn and remember the spellings of new words that they encounter while reading. Thus, the degree to which a word conforms to the patterns is an important determinant of spelling performance, above and beyond the amount of exposure to the word. Our results also suggest that the position of a letter or letter group plays an important role in memory for words' spellings. Thus, French third graders who encounter a novel item with a doublet in the hard-to-remember medial position sometimes remember that an item contained a doublet but not which letter was doubled. In such cases, they seem to use their knowledge of a graphotactic pattern—that double consonants can occur before but not after single consonants—and this sometimes leads them to transposition errors such as misspelling *guprrane* as *gupprane*. When a doublet is in the salient initial position, in contrast, third graders are better at remembering that the word contained a doublet and where the doublet occurred. Our results confirm that people use their general knowledge about the graphotactic patterns of their writing system, even when other sources of information would logically suffice to produce correct spellings (e.g., Kemp and Bryant, 2003; Pacton et al., 2005; Pacton and Deacon, 2008), and they shed new light on the conditions under which children do this.

### Conflict of interest statement

The authors declare that the research was conducted in the absence of any commercial or financial relationships that could be construed as a potential conflict of interest.

## Appendix

### Stimuli for judgment task

Practice pairs: *vilo-xtyu, rsmt-baso, natu-gnhd*.

Consonants cannot double in word-initial position: *fannous-ffanous, furrois-ffurois, nnifor-niffor, nummar-nnumar, rrafout-raffout, rramin-rammin, ttamir-tammir, ttinot-tinnot*.

Consonants cannot double after a single consonant: *acclomir-acllomir, accriver-acrriver, affrunir-afrrunir, apllover-applover, apprulir-aprrulir, gabiffler-gabifller, mouffrive-moufrrive, rupllave-rupplave*.

Only some consonants can double: *akkoge-attoge, bekkul-befful, boxxit-bottit, onnave-ojjave, oxxile-ommile, tinnas-tihhas, tummet-tukket, ullate-ujjate*.

### Sample story used in experiment 1

Autrefois, les habitants de la campagne se retrouvaient pour le muppile, la fête du village. C'était l'occasion de chanter, de danser et de déguster les spécialités de la région. Au muppile, la coutume était de s'amuser toute la nuit et d'attendre que le jour se lève pour partager un énorme gâteau au nifude. Le nifude est un fruit délicieux que l'on trouvait dans les forêts. Lors d'unmuppile, une drôle d'histoire est arrivée à Loura, une jeune villageoise curieuse et intrépide qui aimait faire des blagues. La fillette a voulu goûter le gâteau avant la fin du muppile parce qu'elle adorait le goût du nifude. Malheureusement, le gâteau se renversa. La pauvre fillette passa le reste de la nuit à chercher la plante qui donne le nifude, pour que sa mère prépare un autre gâteau. Finalement, personne ne se rendit compte de rien et le muppile se termina comme prévu. Le nifude fit encore beaucoup d'heureux.

(In the past, the people of the country met for a muppile, the party of the village. It was an opportunity to sing, dance, and savor the specialties of the area. At the muppile, people would play all night and wait for dawn in order to share a big cake made with nifude. Nifude is a delicious fruit that is found in forests. During a muppile, something funny happened to Loura, a young, curious, and intrepid villager who loved playing jokes. This young girl wanted to taste the cake before the end of the muppile because she loved the taste of nifude. Unfortunately, the cake fell over. The poor girl spent the rest of the night looking for the plant that provides the nifude so that her mother could make a new cake. In the end, nobody noticed anything and the muppile finished as anticipated. The nifude made a lot of people happy).
